# Relation of arterial stiffness to left ventricular structure and function in healthy women

**DOI:** 10.1186/s12947-018-0139-6

**Published:** 2018-09-25

**Authors:** Jing Zhang, Philip J. Chowienczyk, Tim D. Spector, Benyu Jiang

**Affiliations:** 1 Department of Ultrasound, Union Hospital, Tongji Medical College, Huazhong University of Science and Technology, Wuhan, 430022 China; 2 Hubei Province Key Laboratory of Molecular imaging, Wuhan, China; 3 King’s College London British Heart Foundation Centre, Department of Clinical Pharmacology, St. Thomas’ Hospital, Lambeth Palace Road, London, SE1 7EH UK; 4 Department of Twin Research and Genetic Epidemiology, King’s College London, St. Thomas’ Hospital, London, UK

**Keywords:** Arterial stiffness, Left ventricular hypertrophy, Left ventricular twist, Speckle tracking echocardiography

## Abstract

**Background:**

Interactions between the left ventricular (LV) and the arterial system, (ventricular-arterial coupling) are key determinants of cardiovascular function. However, most of studies covered multiple cardiovascular risk factors, which also contributed to the morphological and functional changes of LV. The aim of this study was to examine the relationship between arterial stiffness and LV structure and function in healthy women with a low burden of risk factors.

**Methods:**

Healthy women from the Twins UK cohort (*n* = 147, mean age was 54.07 ± 11.90 years) were studied. Arterial stiffness was evaluated by carotid-femoral pulse wave velocity (cf-PWV). LV structure and function were assessed by two-dimensional speckle tracking echocardiography.

**Results:**

cf-PWV was significantly associated with most measures of LV geometry and function, including relative wall thickness (RWT), E/e’ ratio, global circumferential and radial strain, apical rotation and LV twist (each *p* <  0.05), but bore no relation to global longitudinal strain. After adjustment for age, body mass index, blood pressure and heart rate, cf-PWV was significantly correlated with RWT, global circumferential strain, apical rotation and LV twist (β = 0.011, − 0.484, 1.167 and 1.089, respectively, each *p* ≤  0.05).

**Conclusions:**

In healthy women with a low burden of risk factors, elevated arterial stiffness was intimately interwoven with increased LV twisting even before LV dysfunction becomes clinically evident.

## Background

Arterial stiffening and ventricular remodeling are considered features of cardiovascular ageing [1]. However, the extent to which ventricular remodeling and alteration in ventricular function may be secondary to arterial stiffening is uncertain. Previous studies suggested that aortic stiffening is associated with an increased risk for heart failure with preserved ejection fraction, even in the absence of cardiovascular risk factors [2, 3]. Various associations between arterial stiffness and left ventricular (LV) structure and function have been demonstrated. However, most of these studies were conducted in subjects with multiple cardiovascular risk factors, such as hypertension, diabetes, aortic stenosis or obesity [4–6] that may independently influence LV and arteries. Therefore, studies are warranted to examine the relationship between arterial stiffness and LV structure and function in healthy populations without cardiovascular risk factors.

Although, several echocardiographic studies have shown the relationship between arterial stiffness and LV diastolic dysfunction in healthy populations [1, 3], however, reports regarding the relationship between arterial stiffness and left ventricular myocardial deformation (stain and twist) in healthy populations are scarce. In the present study we examined the relationship between the arterial stiffness and LV structure, diastolic function, systolic stain and twist by using two-dimensional speckle tracking echocardiography in the healthy women with low burden of cardiovascular risk factors from the Twins UK cohort.

## Methods

### Study population

The study population was comprised of a sub-sample of 200 consecutively recruited women from the Twins UK cohort with characteristics similar to the general U.K. population [7] who participated in a longitudinal study on cardiovascular ageing. Women with risk factors requiring treatment, known ischemic heart disease, atrial fibrillation or significant valvular heart disease and those in whom adequate image quality could not be obtained were excluded, leaving 147 healthy women. Haemodynamic measurements were performed in a quiet temperature-controlled laboratory (22 °C to 24 °C). The study was approved by St. Thomas’ Hospital Research Ethics Committee and written informed consent was obtained from all subjects.

### Blood pressure and carotid-femoral pulse wave velocity (cf-PWV)

Brachial blood pressure was measured in duplicate, by using a validated oscillometric monitor (Omron 705 CP, Omron, Tokyo, Japan) after subjects had rested in a supine position for at least 10 min. cf-PWV was measured using sequential carotid-femoral ECG-referenced applanation tonometry using the SphygmoCor system (Atcor Medical, Sydney, Australia). The path distance between the carotid and femoral sites was estimated from the distance between the sternal notch and femoral artery at the point of applanation. At least two sequential measurements satisfying the inbuilt quality control criteria of the SphygmoCor system were obtained and the mean value was used for analysis.

### Echocardiography

A transthoracic echocardiographic examination was performed in the left lateral decubitus position using a Vivid-7 ultrasound scanner with an M3S cardiac probe (General Electric Healthcare, Milwaukee, WI, USA). Two-dimensional views were obtained via the apical (4-, 2-chamber, and long-axis views) and parasternal (short-axis views at mitral valve, papillary muscle, and apical levels) approaches. Three consecutive cardiac cycles of each view were acquired during end-expiration breath holding and stored digitally on a hard disk for offline analysis. All images were obtained at a frame rate of 50 to 80 frames per second. Time of aortic valve closure was assessed by aortic valve motion in the apical long-axis view.

Echocardiographic measurements were obtained offline on a PC work-station by using a commercially available analysis software package (EchoPAC, version 11, GE Healthcare, Norway). LV end-diastolic dimension (LVED), end-systolic dimension (LVES), end-diastolic septal thickness (IVSd) and end-diastolic LV posterior wall thickness (LVPWd) were measured from a parasternal long-axis view in accordance with recommendations of the American Society of Echocardiography [8]. LV mass (LVM), LV mass index (LVMI) and relative wall thickness (RWT) were calculated according to standard formula. LV end-systolic, end-diastolic volumes and LV ejection fraction (LVEF) were measured using the biplane Simpson’s method.

Transmitral flow velocity was obtained from the apical 4-chamber view using pulsed Doppler. The peak early diastolic velocity (E), peak atrial systolic velocity (A), and E/A ratio were measured. Peak motion velocity in early diastolic (e’) and atrial contraction (a’) were measured by tissue Doppler imaging (TDI) at all basal segments from apical 4- and 2-chamber views. Measurements were averaged over all segments.

### Two-dimensional speckle tracking echocardiography

Two-dimensional speckle tracking imaging was performed on three apical views and parasternal short-axis views of the LV. The endocardial border of the LV was defined by placing several points along it and the width of interest was then adjusted to accommodate myocardial thickness. The image analysis software identified natural acoustic markers that moved with the tissue. Automatic frame-by-frame tracking of these markers during the cardiac cycle yielded a strain value and a strain rate. Each LV view was divided into 6 equal segments, providing strain and strain rate curves for these segments.

Global longitudinal strain (GLS) measurements were obtained on 3 apical views. The global circumferential and radial strain (GCS, GRS) measurements were acquired on the short-axis view at papillary muscle level. The systolic peak of strain values between aortic valve opening and closing was determined. Rotation parameters were measured in short-axis views at mitral valve level (basal rotation, BaseRot) and apical level (apical rotation, ApexRot). Basal-to-apical LV twist was defined as the net difference between apical and basal rotations at the aortic valve closing. Intra- and inter-observer variability of LV strain, rotation and twist were measured in 15 subjects.

### Statistical analysis

Statistical analyses were performed using SPSS (version 16.0, SPSS, Inc., Chicago, Illinois). Continuous variables were presented as means ± standard deviation (SD). The sample was divided into 3 groups according to tertiles of the distribution of cf-PWV. Differences between groups were evaluated by one-way analysis of variance (ANOVA). Bivariate correlations between cf-PWV and LV structural and functional measurements were assessed by Pearson’s correlation coefficient. Multivariate linear regression analysis was then applied to examine the independence of association between cf-PWV and LV measurements after adjustment for age, BMI, blood pressure (systolic blood pressure and pulse pressure) and heart rate. Intra- and inter-observer variability of LV strain and twist were assessed using intra-class correlation coefficients (ICC) with 95% confidence intervals and coefficient of variation (expressed as the mean ± standard deviation of the absolute differences between the two measurements divided by the mean value (%)). A *p*-value < 0.05 was considered statistically significant and all tests were 2-tailed.

## Results

**Fig. 1:**
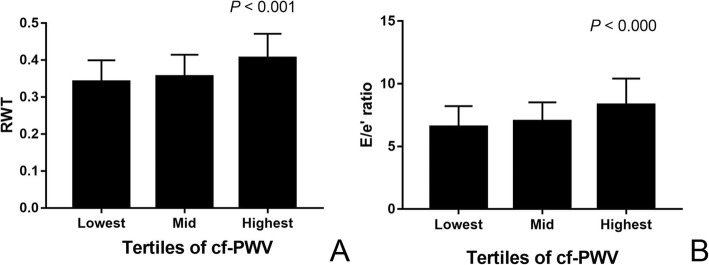
Comparison of RWT (**a**) and E/e’ (**b**) in the population subdivided by tertile of cf-PWV

Demographic and clinical characteristics of the subjects are summarized in Table 1. Mean age was 54.1 ± 11.9 years, mean brachial systolic/diastolic blood pressure were 122 ± 12.5/75 ± 8.8 mmHg and mean cf-PWV was 8.70 ± 1.48 m/s. Echocardiographic parameters are shown in Table 2. To examine the association of arterial stiffness with LV structure and function, subjects were divided into 3 tertiles according to their cf-PWV. RWT, E/e’, GCS, ApexRot and LV twist rose with increasing cf-PWV across the groups (Fig. 1), whereas the E/A ratio and e’/a’ ratio showed the opposite change. LVED, LVES, LVMI, LVEF, GRS, GLS and BaseRot were all similar among the 3 groups.

**Table 1:** Demographic and clinical characteristics of the study subjects (*n* = 147)

Variable	Mean ± SD
Age, y	54.07 ± 11.90
Height, cm	162.31 ± 6.74
Weight, Kg	64.74 ± 9.55
BMI, Kg/m^2^	24.59 ± 3.44
BSA, m^2^	1.70 ± 0.14
pSBP, mmHg	122.39 ± 12.53
pDBP, mmHg	74.63 ± 8.79
PP, mmHg	47.91 ± 9.21
HR, bpm	61.54 ± 9.20
cf-PWV, m/s	8.70 ± 1.48

*BMI* body mass index, *BSA* body surface area, *pSBP* brachial systolic blood pressure, *pDBP* brachial diastolic blood pressure, *PP* pulse pressure, *HR* heart rate, *cf-PWV* carotid-femoral pulse wave velocity

**Table 2:** Echocardiographic parameters of the study subjects (*n* = 147)

Variable	total (*n* = 147)	Lowest cfPWV (*n* = 49)	Mid cfPWV (*n* = 49)	Highest cfPW (*n* = 49)	p
Echocardiographic Characteristics					
RWT	0.37 ± 0.07	0.34 ± 0.06	0.36 ± 0.06	0.41 ± 0.07	< 0.001
LVMI, g/m^2^	71.82 ± 13.09	69.71 ± 13.43	70.33 ± 13.66	75.38 ± 11.63	0.084
LVED, cm	4.53 ± 0.39	4.60 ± 0.36	4.55 ± 0.40	4.44 ± 0.39	0.132
LVES, cm	2.97 ± 0.33	3.05 ± 0.31	2.94 ± 0.35	2.92 ± 0.32	0.125
LVEF, %	65.38 ± 5.77	64.50 ± 5.00	65.09 ± 6.10	66.29 ± 5.99	0.479
E/A	1.28 ± 0.37	1.44 ± 0.36	1.28 ± 0.40	1.12 ± 0.29	< 0.001
e’ / a’	1.21 ± 0.41	1.51 ± 0.37	1.23 ± 0.35	0.93 ± 0.28	< 0.001
E/e’	7.29 ± 1.89	6.56 ± 1.63	7.02 ± 1.50	8.30 ± 2.11	< 0.000
2D Speckle-Tracking Imaging					
GCS, %	−22.95 ± 2.81	− 21.92 ± 2.40	−23.08 ± 2.86	−23.75 ± 2.85	0.009
GRS, %	42.86 ± 14.64	45.82 ± 13.32	41.96 ± 13.81	40.97 ± 16.33	0.260
GLS, %	−21.51 ± 1.65	−21.73 ± 1.64	−21.78 ± 1.89	−21.84 ± 1.41	0.955
BaseRot, °	−5.91 ± 3.27	−5.22 ± 3.72	−5.96 ± 2.59	−6.50 ± 3.38	0.192
ApexRot, °	9.85 ± 4.90	9.08 ± 4.95	9.06 ± 4.72	11.30 ± 4.78	0.046
Twist, °	15.72 ± 5.50	14.24 ± 4.81	14.97 ± 4.83	17.78 ± 6.14	0.006

The data are expressed as mean ± SD

*RWT* relative wall thickness, *LVMI* left ventricular mass index, *LVEF* left ventricular ejection fraction, *E* peak early diastolic transmitral flow velocity, *A* peak atrial systolic transmitral flow velocity, *e’* peak early diastolic mitral annular motion velocity, *a’* peak atrial systolic mitral annular motion velocity, *GCS* global circumferential strain, *GRS* global radial strain, *GLS* global longitutide strain, *BaseRot* rotation at basal level, *ApexRot* rotation at apical level

°Degree

**Fig. 2:**
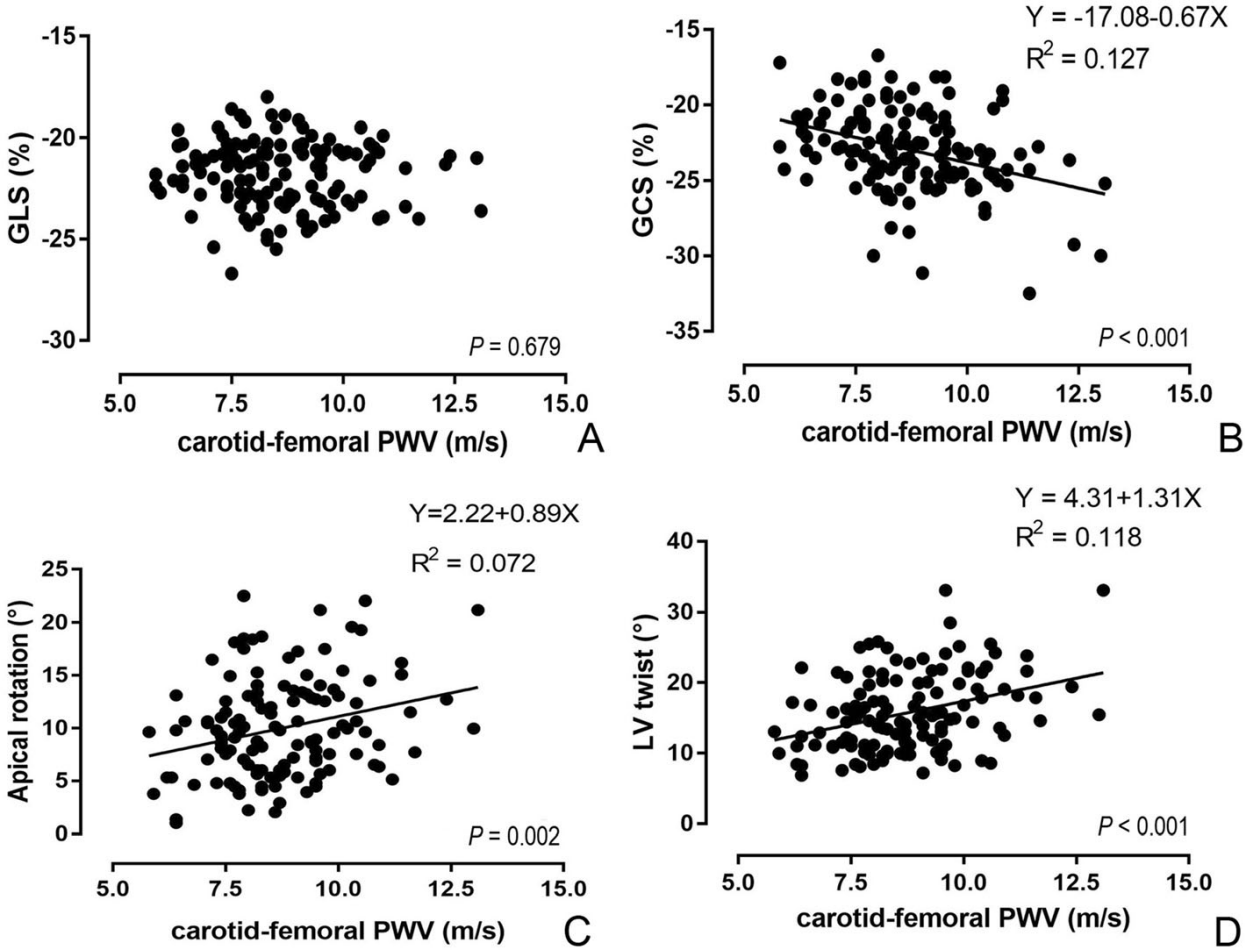
Linear correlation of cf-PWV with the GLS (**a**), GCS (**b**), ApexRot (**c**) and LV twist (**d**)

Pearson’s correlation analysis showed that cf-PWV was correlated with most measures of LV geometry and function, including RWT, LVED, LVES, E/A, e’/a’, E/e’, GCS, GRS, ApexRot, and LV Twist, but bore no significant relation to LVMI, LVEF, GLS and BaseRot. Figure 2 demonstrated the relationship of cf-PWV to the parameters of LV strain and rotation. However, multivariable linear regression revealed that, when adjusted for age, BMI, systolic blood pressure, pulse pressure and heart rate, cf-PWV was only significantly correlated with RWT, GCS, ApexRot and LV Twist (β = 0.011, − 0.484, 1.167 and 1.089, respectively, each *p* <  0.05, Table 3).

**Table 3:** Bivariate correlation and multivariate linear regression between cf-PWV and LV parameters

Echo parameters	Univariate analysis^a^		Multivariate analysis^b^	
	r (95%CI)	*p* value	β (95%CI)	*p* value
LV Structure				
RWT	0.469 (0.329, 0.589)	< 0.001	0.011 (0.001, 0.020)	0.030
LVMI, g/m^2^	0.165 (−0.006, 0.327)	0.059	−0.920(−3.094, 1.254)	0.404
LVED, cm	−0.174 (− 0.338, − 0.014)	0.044	−0.047 (− 0.108, 0.015)	0.134
LVES, cm	−0.212 (− 0.356, − 0.036)	0.014	−0.027 (− 0.081, 0.028)	0.336
Diastolic Function				
E/A	- 0.339(−0.483, − 0.178)	< 0.001	0.030 (− 0.023, 0.084)	0.258
e’ / a’	- 0.574 (− 0.680, − 0.446)	< 0.001	−0.016 (− 0.061, 0.028)	0.469
E/e’	0.434 (0.282, 0.566)	< 0.001	0.094 (−0.153, 0.342)	0.453
Systolic Function				
LVEF, %	0.158 (−0.049, 0.353)	0.134	–	–
GCS, %	- 0.356(−0.500, − 0.198)	< 0.001	− 0.484 (− 0.939, − 0.030)	0.037
GRS, %	- 0.206 (− 0.363, − 0.037)	0.017	−2.316 (− 4.900, 0.268)	0.078
GLS, %	- 0.037 (− 0.208, 0.136)	0.679	–	–
BaseRot, °	- 0.159 (− 0.322, 0.015)	0.072	0.082(− 0.476, 0.641)	0.771
ApexRot, °	0.276 (0.108, 0.428)	0.002	1.167 (0.354, 1.980)	0.005
Twist, °	0.343 (0.181, 0.487)	< 0.001	1.089 (0.193, 1.985)	0.018

^a^ Pearson’s correlation analysis

^b^ models adjusted for age, BMI, pSBP, PP, HR.

°Degree

There was a good reproducibility and consistency in the measurement of the LV strain and twist. Detailed inter-observer and intra-observer variability are shown in Table 4.

**Table 4:** Interobserver and Intraobserver variability

Variable	Interobserver		Intraobserver	
	ICC(95% CI)	CoV(%)	ICC(95% CI)	CoV(%)
GLS	0.896(0.709–0.965)	4.05 ± 2.97	0.895(0.725–0.962)	6.31 ± 4.78
GRS	0.891(0.695–0.964)	18.19 ± 10.72	0.891(0.708–0.962)	15.05 ± 7.51
GCS	0.902(0.723–0.964)	10.93 ± 6.28	0.914(0.771–0.969)	11.89 ± 8.79
BaseRot	0.893(0.687–0.966)	17.38 ± 11.60	0.913(0.760–0.970)	18.87 ± 10.94
ApexRot	0.910(0.734–0.972)	10.93 ± 5.17	0.925(0.815–0.971)	12.02 ± 7.62
Twist	0.921(0.762–0.975)	13.22 ± 11.15	0.959(0.896–0.984)	11.91 ± 7.69

*ICC* intraclass correlation coefficients, *CoV* coefficient of variation;

## Discussion

Arterial stiffening is the hallmark of vascular ageing and, when measured by cf-PWV, is predictive of outcome independent of blood pressure components. cf-PWV is a major determinant of the pulsatile load on the ventricle up to the time of peak, myocardial wall stress and thus may have an effect on ventricular remodeling and ventricular function that is not captured by classical blood pressure components [9, 10].

Consistent with previous results [11–15], this study demonstrated cf-PWV to be significantly positively correlated with RWT after adjustment for confounding factors, such as age and blood pressure, thereby supporting the hypothesis that elevated aortic impedance exerts a direct effect on LV concentric remodeling (i.e., increased RWT) in healthy women. With progressive stiffening of the elastic arteries, LV afterload increases because the reflected waves return earlier during systole, thus, leading to myocardial hypertrophy.

Currently, the noninvasive estimation of LV diastolic function is based on conventional echocardiographic and tissue Doppler measurements. LV diastolic dysfunction is mainly characterized by decreased E/A ratio, e’/a’ ratio, and enhanced E/e’ ratio. Arterial stiffness has been found to be associated with diastolic dysfunction in various populations [2, 4, 16, 17]. However, our study showed that after adjustment for confounding factors, there existed no significant correlation between cf-PWV and LV diastolic dysfunction in women with a low burden of risk factors. This discrepancy may be explained by demographic differences of study subjects since the subjects in this study were younger and healthier with narrow PP and lower cf-PWV. In addition, other confounding factors, such as smoking habits, alcohol consumption, physical activity were not taken into account in our study but might also be contributors.

Results of previous studies on the relationship of arterial stiffness to LV systolic function have varied substantially according to the methods used and populations studied. Arterial stiffness has been associated with sub-clinical LV systolic dysfunction and impaired LV longitudinal function in patients with hypertension [18], type 1 diabetes mellitus [6], and in a general population [19]. However, Myung et al. [20]*.* found carotid artery stiffness parameter (β) not to correlate with LV systolic function in hypertensive patients.

LV strain and twist, due to their complicated helical myocardial fiber architecture, play important roles in ventricular systolic and diastolic performance. To understand the progression of LV systolic dysfunction at different stages of heart disease, it is important to analyze the various components involved in LV deformation. Recently, the angle-independent two-dimensional speckle tracking echocardiography has been successfully used for the measurement of myocardial deformational and rotational parameters. This technique tracks the motion of speckles within the myocardium, allowing complete and accurate assessment of myocardial deformation in all three spatial dimensions. In the present study, we measured LV longitudinal, radial, and circumferential strain and LV twist in healthy women by using speckle tracking echocardiography and found that cf-PWV bore no significant relation to LV longitudinal strain and basal rotation. However, there was a significant positive correlation between cf-PWV and circumferential strain, apical rotation and LV twist after adjustment for confounding factors. Consistent with our findings, previous studies also revealed an increase in LV twisting with increasing aortic stiffness in patients with diabetes, hypertension, and diastolic heart failure [11, 21]. The precise mechanisms underlying increased LV twist remain unclear. LV twisting and rotational deformation are directly related to right- and left-handed helical orientations of LV myocardial fibers. Some investigators explained that during systole, counterclockwise rotation of the LV apex, is dictated mainly by the subepicardial fibers, generating high torque and dominates LV torsional forces. The clockwise rotation of the LV base is mainly determined by the subendocardial fibers, which may be vulnerable to increased cf-PWV and associated increased myocardial pressure load and oxygen consumption, resulting in subendocardial injury [22–26]. Such injury may accentuate both LV apical rotation and twist as the subepicardial torque is left unopposed.

The discrepancies between our findings and previous studies about the relationship between arterial stiffness and LV GLS may be explained by a relatively low cf-PWV (mean cf-PWV was 8.85 ± 1.75 m/s) in our subjects and certain confounding factors, such as an effect of cardiovascular risk factors on the systolic function.

Our results showed that increased arterial stiffness might contribute to increased LV twist in healthy women with a low burden of risk factors. Given that LV twist is an important determinant of LV function and the change in twist always far precedes irreversible tissue damage [27, 28], it can be postulated that increased LV twist might be a compensation for injured sub-endocardium, thereby preserving cardiac contractility. Furthermore, the change in LV twist may serve as an indicator for early LV dysfunction, even before LV diastolic dysfunction or LV GLS depression becomes clinically evident. On the other hand, our study showed a close interaction between cf-PWV and LV twist, thus cf-PWV may be a good tool to assess sub-clinical LV dysfunction, especially when it is used in healthy populations with a low burden of risk factors. More importantly, improving cf-PWV may be a good therapeutic approach to stop the progression of LV dysfunction or to prevent the development of heart failure with preserved ejection fraction. However, further multicenter and prospective studies are required to confirm our hypothesis.

### Limitations

The study had some limitations. First, the subjects were limited to female twins, although their lifestyle was similar to the general population of women in the U.K. The applicability of our findings to men or other ethnicities, remains to be confirmed in future studies. Second, our study is cross-sectional, therefore observational nature and causal relationship cannot definitively be established. This is particularly relevant as some of the observed correlations are likely to be bidirectional. In addition, effects of other confounding factors, such as smoking habits, alcohol consumption and physical activity could not be eliminated. Further longitudinal studies are warranted. Third, the results of this study need to be further confirmed by large sample size studies. Moreover, several unproven assumptions are involved in assessment of the relationship between the arterial stiffness and LV dysfunction. Finally, we only used parasternal short-axis view at the level of the papillary muscle to evaluate GRS and GCS, and the exact locations of the basal and apical planes varied from individual to individual, which might cause intrinsic error in the calculation of LV twist.

## Conclusions

This study demonstrates that in healthy women with a low burden of risk factors, increased aortic stiffening is associated with an increase in LV twist but is not significantly related to LV diastolic function and global longitudinal strain of LV myocardium. These findings suggest a close interaction between arterial remodeling and LV twisting even before LV dysfunction becomes clinically evident.

## Abbreviations

A: Peak atrial systolic transmitral flow velocity
a’: Peak atrial systolic mitral annular motion velocity
ApexRot: Rotation at apical level
BaseRot: Rotation at basal level
cf-PWV: carotid-femoral pulse wave velocity
E: Peak early diastolic transmitral flow velocity
e’: Peak early diastolic mitral annular motion velocity
GCS: Global circumferential strain
GLS: Global longitudinal strain
GRS: Global radial strain
IVSd: End-diastolic septal thickness
LV: Left ventricular
LVED: LV end-diastolic dimension
LVEF: LV ejection fraction
LVES: LV end-systolic dimension
LVM: LV mass
LVMI: LV mass index
LVPWd: End-diastolic LV posterior wall thickness
RWT: Relative wall thickness
TDI: Tissue Doppler imaging
